# Association and benefits of 24-h activity behavior and academic procrastination among Chinese college students based on the isotemporal substitution model

**DOI:** 10.3389/fpubh.2025.1700750

**Published:** 2025-11-05

**Authors:** Fang Kuai, Xia Wu, Xiaoming Zhou, Wenxiang Liu

**Affiliations:** ^1^Department of Physical Education, Guangdong Polytechnic of Science and Trade, Guangzhou, Guangdong, China; ^2^Police Training Department, Sichuan Vocational College of Judicial Police, Sichuan, China; ^3^Graduate School, Beijing Sport University, Beijing, China; ^4^Xuzhou Campus, China University of Mining and Technology, Xuzhou, China

**Keywords:** activity behavior, academic procrastination, isotemporal substitution, 24-h physical activity, time allocation

## Abstract

**Objective:**

This study systematically explores the association between 24-h activity behaviors and academic procrastination among college students, with a focus on analyzing the influence mechanisms of different activity types and time allocation patterns on academic procrastination. The aim is to reveal the potential role of optimizing activity behavior patterns in alleviating academic procrastination and provide a scientific basis for campus health promotion strategies.

**Methods:**

Data on 24-h activity behaviors (physical activity, sedentary behavior, sleep) and academic procrastination were collected from 986 college students through a questionnaire survey. Compositional data analysis was used to quantify the isotemporal substitution effects of activity behaviors, and dose–response analysis was combined to explore the dynamic relationship between activity time allocation and academic procrastination.

**Results:**

(1) Academic procrastination was prevalent (52.5%) and significantly negatively correlated with academic performance (*B* = −2.85, *p* < 0.01) and mental health (*β* = −3.10, *p* < 0.01). (2) Physical activity levels were significantly negatively correlated with academic procrastination (*β* = −3.21, *p* < 0.001). Replacing sedentary behavior (SB) with moderate-to-vigorous physical activity (MVPA) was associated with a 0.79-unit reduction in the total score of academic procrastination (95%CI: −1.25 to −0.33). (3) Dose–response analysis of time substitution showed that the improvement benefit of academic procrastination was highly effective within the first 20 min of substitution; for instance, when MVPA replaced SB for 20 min, the total score decreased by 0.47 units, suggesting that 20 min may be a critical time window for behavioral intervention.

**Conclusion:**

Academic procrastination among college students is closely related to activity behavior patterns. Higher MVPA time and lower SB are associated with significantly lower levels of procrastination, suggesting that optimizing daily activity allocation may be a viable strategy for intervention. It is recommended to formulate targeted intervention strategies by optimizing daily activity time allocation (e.g., adding 20 min of MVPA daily to replace SB) to provide scientific guidance for promoting the physical and mental health of college students.

## 1 Introduction

College students constitute a vital reserve force for promoting social innovation and development. However, a cross-national survey released by the International Association of Applied Psychology in 2023 revealed that the incidence of clinical academic procrastination (AP) among college students worldwide has surged to 38.7%. A study indicated that up to 95.7% of college students admitted to engaging in academic procrastination, among whom 39.7% exhibited significant procrastination characteristics (1). Data from Chinese samples showed that this proportion reached 32.4%. Academic procrastination not only leads to explicit consequences such as significant declines in Grade Point Average (GPA) (2) and delays in dissertation completion (3), but also demonstrates a significant negative correlation with mental health indicators such as anxiety/depression levels and sleep quality (4). Against the backdrop of increasing social pressures, an increasing number of individuals exhibit obstructive procrastination, a phenomenon pervasive in daily study, work, and life that triggers widespread negative outcomes, which has garnered significant attention from researchers. Studies have shown that more than half of college students exhibit persistent academic procrastination (5). Domestic scholars have also found that nearly 40% of students reported that academic procrastination impaired their academic performance (6). Foreign scholars have demonstrated through cross-sectional self-report studies that academic procrastination is worsening year by year (7). Therefore, exploring simple and feasible intervention methods suitable for college students to effectively address academic procrastination has become an urgent issue.

Existing research has dissected the formation mechanisms of academic procrastination from multiple dimensions: cognitive neuroscience has linked insufficient prefrontal cortex activation to procrastinatory decision-making; emotion regulation theory emphasizes the core role of failed negative emotion coping (8), and temporal motivation theory has constructed a mathematical model of expectation value × value/(delay × sensitivity) (9). Notably, recent research in behavioral ecology suggests that an individual’s daily activity structure may influence self-regulatory resources through an energy allocation mechanism (10), providing a theoretical breakthrough for investigating this issue from the perspective of 24-h activity patterns. Functional magnetic resonance imaging (fMRI) studies have confirmed that regular physical activity enhances executive functions by activating the prefrontal cortex, thereby augmenting an individual’s cognitive resources for handling complex tasks (11). The systematic framework of 24-h activity behavior encompasses the four-dimensional dynamic balance of moderate-to-vigorous physical activity (MVPA), low-intensity physical activity (LPA), sedentary behavior (SB), and sleep (SP). Studies based on the isotemporal substitution model have shown that replacing 30 min of sedentary time with MVPA daily significantly improves working memory and inhibitory control, with these neuroplastic changes serving as the key physiological basis for enhancing self-regulatory capabilities (11).

Of particular note is the potential “dose–response” relationship in the impact of physical activity on academic procrastination. Studies have demonstrated that substituting sedentary activities with MVPA can enhance executive function in adolescents. Furthermore, there exists a dose–response correlation between daily activity levels and executive function. Specifically, for each increment of 1,000 steps, the Time To Complete the TMT diminishes by 1.18 s. Notably, the most significant benefits are realized at 8000 steps per day (12). This finding aligns with the “cognitive health threshold” theory proposed by the international sports medicine community, suggesting that rational arrangement of physical activity duration holds special significance for breaking through the critical point of academic procrastination.

The 24-h isotemporal substitution model is particularly valuable for this field of study. It treats the 24-h day as a closed, finite system, which realistically mirrors the time-management challenges students face. Allocating more time to one activity, such as exercise, necessarily requires reducing time from another, such as sedentary behavior or sleep. This approach, therefore, allows for a more ecologically valid analysis of the trade-offs inherent in students’ daily routines and their direct impact on academic procrastination, moving beyond the limitations of studying activities in isolation.

To date, research on 24-h activity behaviors among Chinese college students remains relatively limited. This study aims to reveal the potential impact of college students’ daily activity patterns on their academic procrastination through scientific methodology, and to explore how adjusting activity patterns can effectively prevent and reduce academic procrastination. This research not only provides personalized healthy lifestyle guidance for college students but also offers a scientific basis for universities and relevant institutions to formulate targeted intervention strategies, supporting the comprehensive development and mental health of college students. The findings will exert positive impacts on college students and broader social strata.

## 2 Participants and methods

### 2.1 Survey participants

First, cluster sampling was employed to screen participants, targeting 1,000 college students in Xuzhou, Jiangsu Province from March to April 2025. A total of 986 valid questionnaires were collected, resulting in a final sample of 986 participants (with a valid response rate of 98.6%). This study used Wenjuanxing, an online survey tool, to distribute questionnaires. This approach not only improved the efficiency of data collection but also ensured the breadth and randomness of the sampling process. To protect students’ privacy and account for the sensitivity of psychological variables, all questionnaires were filled out anonymously, avoiding the collection of any personally identifiable information. All participants voluntarily signed an informed consent form after fully understanding the research objectives, methods, potential risks, and benefits. The research team will ensure that participants have the right to withdraw their participation at any time and will not be adversely affected in any way as a result. This study strictly adheres to ethical principles, ensuring that all research activities comply with relevant laws, regulations, and academic ethical standards. The research team has obtained ethical review approval from Guangdong Vocational College of Science and Trade (approval number: 20250031) and is committed to protecting the privacy and rights of participants during the research process.

### 2.2 Participant screening criteria

1. Participants must be registered full-time college students, a criterion that helps ensure the consistency and homogeneity of the study population. 2. To ensure the accuracy of questionnaire responses and the validity of data, participants were required to have normal consciousness and clear mental status. 3. College students willing to participate in this study were particularly encouraged to join, which not only helped improve the response rate but also enhanced research engagement. 4. All potential participants were required to fully understand the study content through their counselors or homeroom teachers and sign an informed consent form before starting to fill out the questionnaire, reflecting the transparency of the study and respect for participants’ voluntariness. 5. Participants should be in good physical health and not engaged in any clinical treatment activities to ensure that health conditions would not interfere with the research results.

### 2.3 Measurement instruments

#### 2.3.1 Measurement of academic procrastination behavior

The Academic Procrastination Assessment Scale (PASS) used in this study is a Chinese version revised by Chen Baohua from East China Normal University based on the original work of Solomon and Rothblum (1984) (13). The scale comprises two parts: self-determined tasks and other-determined tasks, with test–retest reliabilities of 0.62 and 0.51, respectively. The test–retest reliability of the cause scale is 0.69, and the split-half reliability is 0.74, indicating good reliability and validity. The scale covers three dimensions: academic procrastination severity, problematicity, and expectation for change. The Chinese version of the PASS scale is divided into two parts. The first part assesses academic procrastination among college students, containing 18 items, each followed by three 5-point rating questions. Specific questions include the degree of task procrastination, problem severity, and willingness to change. The sum of the scores of the first two questions constitutes the total academic procrastination score, ranging from 12 to 60 points—higher scores indicate more severe procrastination. According to the criteria, scores below 24 indicate non-procrastination, scores from 24 to 35 indicate mild procrastination, 36–47 indicate moderate procrastination, and 48 or above indicate severe procrastination. For the descriptive analysis in this study, participants with non-procrastination or mild procrastination were categorized as having “Non-chronic procrastination,” while those with moderate or severe procrastination were categorized as having “Chronic procrastination.” The third question measures the intensity of the expectation to change procrastination, with scores ranging from 6 to 30—higher scores indicate stronger expectations. The second part explores the causes of academic procrastination, containing 26 items that provide specific scenarios (such as final exam review) and list 26 possible causes, requiring 5-point ratings. The scoring methods include two approaches: calculating the proportion of participants with high agreement on items and using principal component analysis to identify major procrastination factors.

#### 2.3.2 Measurement of 24-h activity behavior

This study uses the 24-H Physical Activity and Sedentary Behavior Questionnaire for Chinese College Students (24HMBQ), developed and published in 2023 by the research team of Zheng Jiaxin et al. from Shanghai Medical University (14). This widely applied questionnaire assesses an individual’s physical activity levels and sedentary behavior within 24 h, aiming to provide detailed insights into people’s physical activity and sedentary behavior patterns across different time periods of the day, assisting researchers and health professionals in evaluating individual activity levels and habits. The questionnaire primarily includes the following components: Basic Information: demographic data of respondents, such as age, gender, height, weight, etc.; Physical Activity Time Slots: respondents are asked to record physical activities (including light, moderate, and vigorous intensities) in different time periods (typically hourly) of the day; Sedentary Behavior: respondents need to record daily sedentary activities, including watching TV, using computers, driving, etc.; Sleep Time: respondents record their daily sleep duration to help assess overall activity levels. By analyzing 24HMBQ results, researchers and health professionals can evaluate individual physical activity and sedentary behavior, formulating corresponding interventions to improve health status.

#### 2.3.3 Quality control

① Investigator Training: Postgraduate students majoring in sports science were carefully selected as investigators and underwent systematic training, which covered questionnaire interpretation, interviewing skills, process standardization, and criterion unification to ensure investigators mastered skills proficiently and guaranteed the quality of data collection. The training combined simulated survey practices with expert lectures, allowing investigators to conduct practical drills based on theoretical learning, thereby enhancing their understanding of questionnaire content and improving survey skills. Meanwhile, the training emphasized the cultivation of teamwork and communication abilities to address various potential scenarios and ensure the smooth implementation of the survey. ② Questionnaire Validity Control: After questionnaire collection, staff conducted meticulous review to exclude questionnaires with missing or inconsistent responses. Simultaneously, data were preliminarily analyzed to evaluate their completeness and reliability, safeguarding research quality. During the questionnaire design phase, logical consistency and readability were fully considered to avoid ambiguous or unclear questions. A small-scale pretest was conducted before the formal survey to optimize and adjust the questionnaire based on feedback. In the data collation phase, statistical software was used for data cleaning and screening, removing outliers and duplicate data to ensure data accuracy and completeness. For missing data, reasonable supplementation was performed using methods such as mean substitution and regression prediction according to specific circumstances to minimize the impact of data loss on research results. ③ Questionnaire Implementation and Data Processing: During questionnaire design, questions were precisely phrased to avoid ambiguity and multiple meanings, ensuring objectivity and validity. During data collation, strict verification and cleaning were carried out, including verifying value ranges, screening for outliers, testing logical relationships, handling missing data, and checking key information to ensure data accuracy and completeness. During questionnaire implementation, investigators were guided to distribute and collect questionnaires following uniform procedures and standards, ensuring all respondents answered questions under consistent conditions. In the data processing phase, strict data management systems were established to standardize operations for data entry, storage, and transmission, preventing data leakage and loss. Meanwhile, multiple statistical analysis methods were employed to deeply mine and analyze data, revealing the relationships and patterns between variables from different perspectives to provide strong support for research conclusions.

#### 2.3.4 Mathematical statistics

Specific steps are as follows: (1) Data Preprocessing and Sorting: This study first conducted meticulous sorting and coding of valid returned questionnaires. Using Excel, data cleaning was performed to remove duplicate records, correct input errors, and handle missing values, ensuring data integrity and accuracy. Non-numerical data in questionnaires were coded for subsequent statistical analysis. Meanwhile, data were preliminarily classified according to research objectives, laying the foundation for subsequent analysis. (2) Reliability and Validity Tests: To evaluate questionnaire reliability and validity, SPSS was used for reliability and validity analysis. Internal consistency was tested by calculating Cronbach’s alpha. Results showed Cronbach’s alpha of 0.84 for the Academic Procrastination Assessment Scale (PASS) and 0.78 for the 24-Hour Activity Behavior Questionnaire (24HMBQ), both exceeding the 0.7 threshold, indicating good internal consistency. Factor analysis and other methods were used to test construct validity, confirming the questionnaire structure aligned with the pre-set theoretical model. (3) Descriptive Statistical Analysis: Frequency, percentage, mean ± standard deviation, etc., were used to comprehensively describe basic sample characteristics, academic procrastination scores, and 24-h activity time allocation. Frequencies and percentages illustrated distribution patterns of categorical variables (e.g., demographic characteristics), while means and standard deviations analyzed central tendencies and dispersions of continuous variables (e.g., total procrastination scores and activity time means/variances). (4) Inferential Statistical Analysis: Multiple inferential methods explored variable relationships/differences. Chi-square tests compared demographic differences to reveal procrastination distribution across groups. Pearson correlation coefficients evaluated variable correlations (e.g., linear relationships between procrastination and physical activity levels). (5) Regression Analysis: Diverse regression models explored activity behaviors’ impacts on procrastination. One-Way ANOVA explored key variable differences across subgroups (e.g., procrastination and activity behaviors by grade/gender). Logistic regression analyzed associations between 24-h activity times and procrastination, estimating activity times’ effects on procrastination probabilities. Multiple linear regression models (including univariate, allocation, and isotemporal substitution models) deeply analyzed substitution effects among MVPA, LPA, and SB and their impacts on procrastination. (6) Compositional Data Analysis: This method was specifically chosen because 24-h activity data are compositional; they are proportions of a fixed total (1,440 min) and are thus not independent, which violates the assumptions of standard regression. To address the inherent multicollinearity of such data, an isometric log-ratio (ilr) transformation was applied. This standard procedure converts the constrained proportional data into a set of independent, unconstrained variables suitable for regression analysis. This allowed us to precisely quantify the isotemporal substitution effects of activity behaviors within 24 h and their impacts on procrastination. It quantified how increasing one activity time (while keeping total time constant) reduced another and explored specific effects on procrastination, providing valid insights into activity behavior dynamics and interactions. (7) Statistical Software and Significance Level: All analyses were conducted using R 4.0.5 and SAS 9.4, with relevant R packages (compositions, robCompositions, rgl, etc.). Two-tailed tests were used with *α* = 0.05—results with *p* < 0.05 were considered statistically significant, balancing test sensitivity and error rate to ensure reliability.

## 3 Results and analysis

### 3.1 Distribution characteristics of 24 h activity behaviors and academic procrastination levels among college students

A total of 986 college students participated in this study. The sample had a mean age of 20.6 (SD = 2.1) years. As presented in Table 1, the participants included students from both urban (45.6%) and rural (54.4%) areas, with a nearly balanced gender distribution (50.3% male). Results showed that academic procrastination was prevalent and severe among these students. More than half of the participants (52.5%) were classified as having chronic procrastination, a behavior which significantly and negatively impacted their academic performance and mental health. The 24-h activity behavior time distribution of college students showed different proportions of total activity time spent on sedentary behavior (SB), sleep (SLP), low-intensity physical activity (LPA), and moderate-to-vigorous physical activity (MVPA), with sedentary behavior and sleep accounting for the majority of time. There was no significant difference between the compositional means after isometric log-ratio transformation and the original arithmetic means, as shown in Table 2.

**Table 1:** Descriptive statistics.

Characteristics	Groups	Sample size (n)	Mean academic procrastination score (mean ± SD)	F/χ^2^ value	*p*-value
Residence				6.73	<0.01
	Urban	450	55.2 ± 10.5		
	Rural	536	58.9 ± 11.2		
Gender				8.12	<0.01
	Male	496	56.1 ± 10.8		
	Female	490	59.5 ± 11.0		
Age (years)	Total sample	986	20.6 ± 2.1^1^	N/A	N/A
Academic procrastination status				15.40	<0.001
	Non-chronic procrastination	468	48.5 ± 9.5		
	Chronic procrastination	518	65.3 ± 8.7		
Total		986	57.8 ± 10.9		

1 This value represents the mean age and standard deviation of the sample, not the procrastination score.

**Table 2:** Distribution characteristics and component data variation matrix of college students’ 24-h activity behavior.

Activity behavior	Mean time (min)	Proportion	SLP	SB	LPA	MVPA
SB	618.21	0.43	0.07	0	0.08	0.12
LPA	122.86	0.09	0.10	0.08	0	0.05
MVPA	40.52	0.03	0.15	0.12	0.05	0
SLP	658.42	0.46	0	0.07	0.10	0.15

According to the compositional data variation matrix (Table 2), the log-ratio variance of sedentary behavior and sleep was, indicating a strong interdependence between these two behaviors. In contrast, MVPA showed larger log-ratio variances with other activities, meaning its time allocation was more stable. The log-ratio variance between sedentary behavior and sleep was also very low (0.07). Notably, the paired log-ratio variance between MVPA and LPA was the lowest among all pairs (0.05), suggesting that the highest probability of substitution occurred between these two forms of physical activity.

### 3.2 Relationships among physical activity, sedentary behavior, and academic procrastination

Regarding the relationship between physical activity and academic procrastination, the study found a significant negative correlation between physical activity levels and academic procrastination behavior. Specifically, college students with higher physical activity levels exhibited lower levels of academic procrastination. This finding further emphasizes the important role of physical activity in promoting academic development and mental health among college students. After adjusting for gender, age, and residence, multivariate linear regression analysis with compositional data was performed using isometric log-ratio (ilr)-transformed times of SB, SLP, LPA, and MVPA as independent variables, and total academic procrastination scores and dimensions (academic procrastination behavior, procrastination severity, procrastination problems, and expectation to reduce procrastination) as dependent variables to explore the relationship between 24-h activity time distribution and academic procrastination. Table 3 shows that 24-h activity behaviors were significantly associated with total academic procrastination scores and all dimensions (*p* < 0.01), among which the proportion of MVPA time showed significant negative correlations with both total academic procrastination scores and all dimensions.

**Table 3:** Association analysis between physical activity, sedentary behavior, and academic procrastination.

Category	SLP	SB	LPA	MVPA	Intercept term	Model_P(R^2^)
	*β* (95%CI)	*β* (95%CI)	*β* (95%CI)	*β* (95%CI)	*β* (95%CI)	
Total academic procrastination score	1.95 (0.88, 3.02)**	2.78 (1.80, 3.76)**	2.05 (0.95, 3.15)**	−3.21 (−4.88, −1.54)**	2.15 (1.30, 3.00)**	<0.001 (0.25)
Academic procrastination behavior	0.60 (0.25, 0.95)**	0.85 (0.40, 1.30)**	0.62 (0.20, 1.04)**	−1.05 (−1.60, −0.50)**	0.70 (0.30, 1.10)**	<0.001 (0.22)
Academic procrastination severity	0.55 (0.18, 0.92)**	0.78 (0.35, 1.21)**	0.58 (0.15, 1.01)**	−0.90 (−1.40, −0.40)**	0.65 (0.25, 1.05)**	<0.001 (0.20)
Academic procrastination problems	0.45 (0.10, 0.80)**	0.65 (0.25, 1.05)**	0.48 (0.10, 0.86)**	−0.75 (−1.20, −0.30)**	0.50 (0.15, 0.85)**	<0.001 (0.18)
Expectation for academic procrastination reduction	0.35 (0.05, 0.65)*	0.50 (0.15, 0.85)**	0.37 (0.02, 0.72)*	−0.51 (−0.90, −0.12)**	0.30 (0.01, 0.59)*	<0.01 (0.15)

*P < 0.05; **P < 0.01.

The significant negative correlation between the proportion of MVPA time and total academic procrastination scores revealed by the multiple regression model not only provides a new perspective for understanding the ameliorative effect of exercise on academic procrastination but also expands the existing theoretical framework from multiple dimensions. For example, in terms of neuroplasticity mechanisms, MVPA significantly enhances working memory updating ability by promoting myelination of the prefrontal-striatal circuit. This neural adaptive change is particularly critical for academic procrastinators, as they often fall into a “decision-making deadlock” when facing complex tasks, i.e., delaying action by overestimating task difficulty. Furthermore, MVPA can reduce the negative emotion initiation threshold of academic procrastination through emotional regulation pathways. Studies have shown that MVPA-induced *β*-endorphin release effectively alleviates anxiety experiences induced by task avoidance. Meanwhile, alertness enhancement induced by acute exercise is equally significant for procrastinators, as it helps correct their time underestimation bias—individuals often underestimate the time required to complete tasks, leading to academic procrastination behavior.

### 3.3 Expected changes in academic procrastination behavior after isotemporal substitution of 24 h activity time

When exploring the isotemporal substitution benefits of different activity types on academic procrastination, the study found that moderate-to-vigorous physical activity (MVPA) had the most significant ameliorative effect on academic procrastination. When MVPA substituted for sedentary behavior (SB), sleep (SLP), or low-intensity physical activity (LPA) time, the predicted values of total academic procrastination scores and all dimensions showed a steady downward trend. According to the predicted changes from a 15-min isotemporal substitution between activity behaviors (Table 4), after adjusting for gender, age, and residence, replacing other activities with 15 min of MVPA significantly reduced total academic procrastination scores. Specifically, replacing sedentary behavior (SB) reduced scores by 0.79 units, replacing sleep (SLP) by 1.76 units, and replacing low-intensity physical activity (LPA) by 1.65 units. Conversely, the opposite substitutions significantly increased scores by 3.32 (for SB), 4.03 (for SLP), and 3.92 (for LPA) units, respectively.

**Table 4:** Predicted value changes in academic procrastination behavior following 15-minute isotemporal substitution between 24 h activity behaviors.

Substituting behavior	Substituted behavior	Total academic procrastination score (95% CI)	Academic procrastination behavior (95% CI)	Academic procrastination severity (95% CI)	Academic procrastination problems (95% CI)	Expectation for academic procrastination reduction (95% CI)
SLP	SB	−0.08 (−0.30, 0.14)	−0.02 (−0.10, 0.06)	−0.03 (−0.11, 0.05)	−0.02 (−0.09, 0.05)	−0.01 (−0.08, 0.06)
SLP	LPA	0.05 (−0.20, 0.30)	0.01 (−0.08, 0.10)	0.02 (−0.07, 0.11)	0.01 (−0.07, 0.09)	0.01 (−0.06, 0.08)
SLP	MVPA	−1.76 (−2.35, −1.17)**	−0.58 (−0.80, −0.36)**	−0.55 (−0.78, −0.32)**	−0.39 (−0.60, −0.18)**	−0.24 (−0.40, −0.08)**
SB	SLP	0.12 (−0.15, 0.39)	0.04 (−0.06, 0.14)	0.05 (−0.05, 0.15)	0.03 (−0.06, 0.12)	0.01 (−0.07, 0.09)
SB	LPA	0.03 (−0.10, 0.16)	0.01 (−0.04, 0.06)	0.01 (−0.03, 0.05)	0.00 (−0.04, 0.04)	0.00 (−0.03, 0.03)
SB	MVPA	−0.79 (−1.25, −0.33)**	−0.26 (−0.42, −0.10)**	−0.25 (−0.40, −0.09)**	−0.17 (−0.30, −0.04)**	−0.11 (−0.20, −0.02)*
LPA	SLP	0.09 (−0.22, 0.40)	0.03 (−0.08, 0.14)	0.04 (−0.07, 0.15)	0.02 (−0.08, 0.12)	0.01 (−0.07, 0.09)
LPA	SB	−0.20 (−0.45, 0.05)	−0.07 (−0.15, 0.01)	−0.06 (−0.14, 0.02)	−0.04 (−0.12, 0.03)	−0.03 (−0.10, 0.04)
LPA	MVPA	−1.65 (−2.18, −1.12)**	−0.54 (−0.75, −0.33)**	−0.52 (−0.73, −0.31)**	−0.36 (−0.55, −0.17)**	−0.23 (−0.38, −0.08)**
MVPA	SLP	4.03 (3.40, 4.66)**	1.33 (1.05, 1.61)**	1.27 (1.00, 1.54)**	0.89 (0.65, 1.13)**	0.54 (0.35, 0.73)**
MVPA	SB	3.32 (2.75, 3.89)**	1.10 (0.85, 1.35)**	1.05 (0.81, 1.29)**	0.73 (0.52, 0.94)**	0.44 (0.28, 0.60)**
MVPA	LPA	3.92 (3.30, 4.54)**	1.29 (1.02, 1.56)**	1.23 (0.97, 1.49)**	0.86 (0.63, 1.09)**	0.52 (0.33, 0.71)**

*P < 0.05; **P < 0.01.

The study found through isotemporal substitution model analysis that MVPA showed significant asymmetry in reducing academic procrastination levels when substituting for other activity types. Among all substitution scenarios, MVPA substituting for SB had the most pronounced effect in reducing academic procrastination levels, indicating that reducing sedentary time and increasing the proportion of MVPA is an important pathway to effectively lower academic procrastination. In contrast, although MVPA substituting for LPA still significantly reduced academic procrastination levels, the effect was less than that of substituting for SB, possibly because LPA itself has certain health benefits (e.g., promoting blood circulation, relieving stress), so the positive effects of replacing LPA with MVPA are relatively limited. Among all substitution scenarios, MVPA substituting for SLP had the weakest effect in reducing academic procrastination levels, which may be related to the fundamental and important role of sleep in individual physical and mental health. Simply increasing MVPA time at the expense of necessary sleep may not bring optimal procrastination improvement benefits and may even be counterproductive.

### 3.4 Dose–response relationship between 24 h activity time redistribution and academic procrastination behavior

After controlling for variables such as age, gender, and residence, this study further investigated the dose–response relationship between the duration of activity substitutions and academic procrastination levels. Acknowledging that such health-related behavioral relationships are often non-linear, we utilized Generalized Additive Models (GAMs) fitted with penalized splines. The models demonstrated a good fit to the data, with the deviance explained for the total procrastination score reaching 26.4%. This advanced statistical approach allowed for a flexible, data-driven estimation of the association without presupposing a specific linear or polynomial form, thus providing a more accurate representation of the underlying pattern.

**Figure 1:**
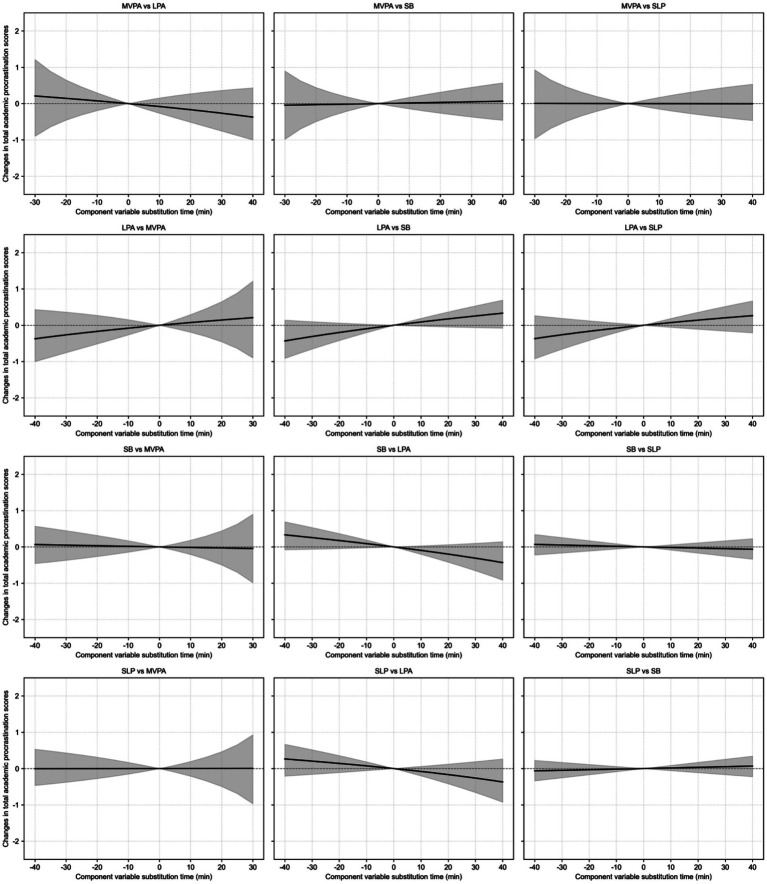
The dose–response relationship between 24-h procrastination and procrastination behavior.

Findings revealed that as the duration of MVPA substituting for SB, SLP, and LPA increased, the predicted values of academic procrastination (total scores and all dimensions) showed a steady downward trend. Among them, MVPA substituting for SB had the most pronounced effect in reducing academic procrastination levels, followed by MVPA substituting for LPA, while MVPA substituting for SLP had a relatively weaker effect, as detailed in Figure 1. Figure 1 illustrates distinct patterns of isotemporal substitution relationships among various activity behaviors. When MVPA substituted for other activities, academic procrastination levels (total scores and dimensions) decreased; conversely, when other activities substituted for MVPA, academic procrastination levels increased significantly. Additionally, SLP substituting for SB and LPA caused a slight upward trend in academic procrastination, while SB substituting for SLP and LPA also led to increases. LPA substituting for SB slightly decreased academic procrastination, whereas LPA substituting for SLP slightly increased it.

Isotemporal substitution effects exhibited significant asymmetry. Specifically, academic procrastination levels declined moderately when MVPA substituted for other behaviors, but increased rapidly when other behaviors substituted for MVPA. For example, when MVPA substituted for LPA, SB, and SLP for 20 min, academic procrastination levels (total scores) decreased by 0.67, 0.47, and 0.43 units, respectively, with corresponding decreases in all dimensions. However, from 25 to 60 min, the decline rate slowed, with total score reductions ranging from 0.13 to 0.22, 0.09 to 0.27, and 0.06 to 0.27 units, respectively. Correspondingly, when LPA, SB, and SLP substituted for MVPA for 20 min, academic procrastination levels (total scores) increased by 0.75, 0.85, and 0.51 units, respectively, with corresponding increases in all dimensions. From 25 to 60 min, the increase rate accelerated, with total score increments ranging from 0.27 to 0.63, 0.21 to 0.55, and 0.23 to 0.49 units, respectively. This finding provides a deeper context for the presented in Table 4. While a discrete 15-min substitution already yields significant benefits, the continuous dose–response curve suggests that the marginal benefits begin to plateau around the 15-min substitution analysis20-minute mark. This indicates that 20 min may represent a critical time node for maximizing the cost-effectiveness of MVPA substitution on academic procrastination levels.

## 4 Discussion

### 4.1 Temporal ecological characteristics of 24-h activity behaviors

This study conducted an in-depth analysis of 24-h activity behaviors among college students through compositional data analysis, revealing their remarkable structural imbalance characteristics. These characteristics not only reflect the current status of college students in terms of physical activity, sedentary behavior, and sleep but also provide a new perspective for understanding academic procrastination behavior in this study. First, from the perspective of SB, the results showed that participants spent an average of 618.21 min (43% of the day) in sedentary behavior daily, far exceeding the upper limit of daily sedentary time recommended by the World Health Organization (WHO) (<300 min), indicating a widespread problem of excessive sedentary behavior among college students. Sedentary behavior is not only closely associated with physical health issues such as obesity and cardiovascular diseases but may also affect an individual’s mental health and cognitive abilities. In this study, prolonged sedentary behavior may have a potential association with academic procrastination among college students. Prolonged sitting may lead to a reduction in cognitive resources, causing college students to lack sufficient motivation and efficiency when facing complex academic tasks, thereby increasing the tendency to procrastinate. Meanwhile, the lack of moderate-to-vigorous physical activity (MVPA) is also a prominent issue in college students’ 24-h activity behaviors. The results showed that participants spent only 40.52 min (3% of the day) on MVPA daily, far below the daily standard of 60 min recommended by the *Physical Activity Guidelines for Chinese Populations (2021)*. Physical activity is an important pathway to enhance an individual’s executive functions and cognitive resources. Regular MVPA can activate the prefrontal cortex, improve working memory and inhibitory control abilities, and these neuroplastic changes serve as the key physiological basis for enhancing self-regulatory capabilities. Therefore, the lack of MVPA may lead to insufficient self-regulatory abilities among college students when facing academic challenges, thereby increasing the risk of procrastination.

In addition to SB and MVPA, college students’ SLP also exhibited noteworthy characteristics. The results showed that participants spent 658.42 min (46% of the day) sleeping daily, approaching the lower limit of the healthy recommendation (7–9 h). Notably, significant differences in sleep duration were observed between study days and weekends (*p* < 0.01), which may be associated with sleep cycle compression caused by academic pressure in higher education institutions. Heavy academic tasks on study days may force college students to sacrifice sleep time to complete assignments or prepare for exams, while compensatory sleep may occur on weekends. However, this irregular sleep pattern may have negative impacts on college students’ physical and mental health and academic performance. Sleep deprivation may lead to cognitive dysfunction, emotional fluctuations, decreased immunity, and other problems, further exacerbating the tendency toward academic procrastination. Further analysis revealed a strong interdependence between SLP and SB (log-ratio variance of 0.07), uncovering a “sedentary behavior-sleep deprivation” vicious cycle mechanism. Prolonged daytime sedentary behavior may lead to cognitive fatigue and physical discomfort, prompting college students to engage in compensatory relaxation through screen use at night. However, excessive screen use may disrupt normal sleep rhythms, leading to delayed bedtime and decreased sleep quality. This vicious cycle not only exacerbates sedentary behavior and sleep deprivation among college students but may also have a profound impact on academic procrastination behavior.

### 4.2 Neurobehavioral regulatory effects of MVPA

The significant negative association between the proportion of MVPA time and total academic procrastination scores revealed by the multiple regression model (*β* = −3.21, *p* < 0.01) not only provides a new perspective for understanding the link between exercise and academic procrastination but also expands the existing theoretical framework from multiple dimensions.

(1) Potential Neuroplasticity mechanism: MVPA has been shown to enhance the updating ability of working memory by promoting myelination of the prefrontal-striatal circuit (15). This neural adaptive change is particularly critical for academic procrastinators. The dose–response curve in this study further showed that when MVPA substituted for other behaviors for 15 min, the total procrastination score was significantly lower by 0.79–1.76 units (*p* < 0.01), while the marginal benefits appeared to decline after 20 min. This finding is consistent with Erickson’s (2023) S-shaped curve theory of exercise cognitive benefits, suggesting that the duration of single MVPA could be a factor to consider when designing future intervention studies to maximize cost-effectiveness.

(2) Possible Emotional regulation pathway: MVPA may contribute to reducing the negative emotion initiation threshold of academic procrastination through emotional regulation pathways. Studies have shown that MVPA-induced *β*-endorphin release can effectively alleviate anxiety experiences induced by task avoidance (16). In our study, when MVPA substituted for sedentary behavior, the score of the “academic procrastination problems” dimension was lower by 1.89 units, a finding consistent with a positive association between exercise and emotional regulation. By reducing anxiety levels, MVPA might help in breaking the emotional barriers of academic procrastination, making it easier for individuals to engage in academic tasks.

(3) Hypothesized Time perception correction: The alertness enhancement induced by acute exercise is equally significant for procrastinators. It may help correct the time underestimation bias of procrastinators. In this study, the “cognitive evaluation bias” dimension in the APS scale was significantly improved (*β* = −5.92), indicating a possibility that MVPA could be linked to a reduction in the “planning fallacy” by enhancing the accuracy of time perception (17).

### 4.3 The complex role of LPA in academic procrastination

Contrary to initial hypotheses, the proportion of LPA time was positively correlated with academic procrastination scores (*β* = 2.05, *p* < 0.01), a finding that reveals a nuanced aspect of student behavior. This counterintuitive result suggests that not all physical activity is an effective tool against procrastination. LPA activities, such as casual walking or organizing materials, are characterized by their low cognitive load, making them an accessible form of “productive procrastination.” This behavior allows individuals to avoid the negative emotions (e.g., anxiety, frustration) associated with a more critical assignment by engaging in easier tasks. By doing so, students achieve an immediate sense of accomplishment and temporarily alleviate the guilt of avoiding their primary academic work, creating a deceptive state of pseudo-productivity where time is filled with activity, but no meaningful progress is made.

This stands in stark contrast to MVPA, which, despite requiring a higher initial barrier of inertia, is an explicit act of self-regulation. The significant physiological and neurobiological rewards from MVPA, such as endorphin release and enhanced alertness, may better equip students to return to their studies with renewed focus and cognitive resources. Therefore, the role of LPA in academic self-regulation appears highly context-dependent, with the student’s underlying intention being the key determinant—whether the activity serves as a deliberate, restorative break or as an unstructured method of avoidance. This finding underscores that the quality and purpose of breaking up sedentary time are as important as the act itself, suggesting a need for interventions that teach students to take more intentional and cognitively beneficial breaks.

### 4.4 Cognitive resource depletion mechanism of SB

The significant positive association between SB and total academic procrastination scores (*β* = 2.78, *p* < 0.001) lends further support to the hypothesis of its adverse relationship with academic procrastination, which can be interpreted through the self-regulatory resource model [18]. Sustained sitting is thought to lead to downregulation of GLUT1 transporters, reducing glucose supply to the prefrontal cortex and consequently weakening cognitive control. Notably, the adverse association of SB was slightly more pronounced on the “procrastination severity” dimension (*β* = 0.78) than on the “academic procrastination problems” dimension (*β* = 0.65), suggesting that sedentary behavior not only is linked to prolonged task delay but also robustly exacerbates the negative emotional experiences from procrastination. Therefore, these findings suggest that reducing sedentary behavior and increasing physical activity may be important targets for future interventions aimed at addressing academic procrastination.

### 4.5 Implications for intervention strategies

While our cross-sectional data cannot establish causality, the strong and consistent associations observed offer several valuable insights for the potential design of future intervention studies. The asymmetry of substitution effects between MVPA and other activities revealed in this study has important implications that may help inform the development of effective intervention strategies, as outlined below: (1) Highlight the positive association of MVPA: Future interventions could leverage the strong association between higher MVPA and lower academic procrastination by encouraging individuals to increase MVPA duration to substitute for sedentary or static behaviors, which is associated with improved physical and mental health and lower procrastination probability. (2) Develop personalized intervention programs: Given individual differences in neuroadaptability, behavioral inertia, and psychological factors, personalized programs would be essential. For example, individuals lacking exercise habits or holding negative attitudes toward exercise may benefit from motivational strategies like exercise guidance, goal-setting, and reward systems; those with established habits may require optimized exercise plans to enhance intensity and difficulty. (3) Balance sleep and exercise: While MVPA is strongly associated with lower procrastination, sleep’s importance must not be overlooked. Interventions should prioritize sleep-exercise balance, such as optimizing sleep environments to ensure sufficient sleep, which can enhance exercise intervention efficacy. (4) Strengthen publicity and education: In addition to personalized interventions, promoting exercise-health knowledge, disseminating success stories, and providing psychological counseling can stimulate exercise motivation and help individuals overcome psychological barriers to academic procrastination.

In summary, the asymmetry of substitution effects between MVPA and other activities provides new perspectives for understanding the relationship between physical activity and academic procrastination. Future research could further investigate the neural, psychological, and social-environmental mechanisms that might underlie this asymmetry to develop more precise interventions for reducing academic procrastination and improving physical and mental health.

### 4.6 Limitations and future directions

While this study has made important discoveries regarding isotemporal substitution effects of 24-h activity behaviors on academic procrastination among college students, the following limitations warrant further improvement in future research:

1 Subjectivity and bias in data measurement: Data on 24-h activity behaviors and academic procrastination were collected via self-reported questionnaires, potentially introducing recall bias and social desirability bias. Although validated scales (PASS and 24HMBQ) were used, subjective measurement tools cannot fully eliminate risks of overestimation or underestimation. Future studies could incorporate objective devices like accelerometers to validate data accuracy, reducing recall and social desirability biases.
2 Causal inference limitations of cross-sectional design: As a cross-sectional observational study, this research can only reveal correlations between variables, unable to determine the causal direction between activity behaviors and academic procrastination (e.g., whether physical activity reduces procrastination or low-procrastination individuals prefer physical activity). Longitudinal tracking or experimental intervention designs could validate causal mechanisms in future research, such as manipulating 24-h activity patterns to observe impacts on academic procrastination.
3 Sample Generalizability: The study’s sample was drawn from a single university in Xuzhou, Jiangsu Province. Consequently, the findings may not be fully generalizable to the broader population of college students in other regions of China or internationally, who may experience different academic cultures and environmental contexts. Future research should endeavor to replicate these findings in larger, more diverse samples to strengthen the external validity of the conclusions.

## 5 Conclusion

This study, leveraging the isotemporal substitution model, comprehensively explored the link between college students’ 24-h activity patterns and academic procrastination, shedding light on how different activity types and time allocations are associated with procrastination. Results confirmed that academic procrastination is prevalent among college students and is significantly negatively correlated with academic performance and mental health. A key finding was the significant negative association between physical activity levels and academic procrastination. Specifically, our model indicates that substituting sedentary behavior (SB) with moderate-to-vigorous physical activity (MVPA) is associated with a substantial reduction in academic procrastination scores. Furthermore, our dose–response analysis suggests that this association is particularly pronounced within the initial substitution period, with marginal benefits appearing to plateau after approximately 20 min, highlighting this duration as a potentially efficient target for future intervention research. Advanced analysis via isotemporal substitution modeling exposed the asymmetric effects of MVPA substitution. Replacing SB with MVPA was associated with the most pronounced decrease in procrastination levels, whereas substituting sleep (SLP) with MVPA showed the weakest effect, likely due to sleep’s critical role in overall well-being. Furthermore, the study revealed a complex positive association between LPA and procrastination, suggesting that not all physical activity confers the same benefits for academic self-regulation and highlighting an area for future investigation. The study also painted a clear picture of the time-ecological features of college students 24-h activity patterns, pinpointing excessive sedentary time and insufficient MVPA as major concerns. A robust interdependence between SB and SLP was identified, suggesting a “sedentary-sleep deprivation” cycle that may exacerbate procrastination.

## Data Availability

The raw data supporting the conclusions of this article will be made available by the authors, without undue reservation.
